# Welfare Work in Jeopardy

**Published:** 1921-06-25

**Authors:** 


### Welfare Work in Jeopardy.

### MUNICIPAL OR OUTSIDE NURSES?

Some interesting points concerning welfare work are contained in a report of the Southwark Council's Health Committee. The Ministry of Health, asked to confirm the appointments of three nurses and a mother's help for maternity and child-welfare work, has intimated that, on the ground of economy, it is not prepared to sanction these appointments at present. At the same time, the Ministry considers that provision should be made for the nursing of infantile infectious diseases, and suggests that the Council should resume negotiations with the District Nursing Association already working in the area, with a view to contracting for the services of their nurses at a fixed payment per visit.

The Medical Officer of Health states that the nursing staff in Southwark comprises three qualified nurses, but these are not concerned in general nursing of persons in the borough. The general nursing is carried out by Benson House and Ranyard nurses. From his long experience of nursing in the borough he is convinced that the nursing of measles and other notifiable diseases, together with the nursing of sick children at the centres, is more efficiently and economically carried out by nurses directly engaged by the Borough Council than by employing outside nursing institutions, and that the former high mortality in cases of measles can be immensely lessened by early and efficient nursing under the direct supervision of the Ministry of Health. Southwark can fully occupy at any time the services of one nurse for measles. With regard to the sick nurses at the centres, this is absolutely necessary if the greatest good is to be obtained from the maternity and child-welfare scheme. What was the good of instituting centres throughout the borough and providing doctors and health visitors unless the developmental ailments of children are attended to by nurses and remedied?

In view of these facts set out by the Medical Officer of Health the Health Committee expressed the opinion that the Council is justified in the appointment of the three nurses and the mother's help. Hence the Committee asks that the Health Ministry should send an inspector to Southwark to report on the need of strengthening the welfare work.

